# Supplementary material for: Community composition and the environment modulate the population dynamics of type VI secretion in human gut bacteria
Source: bioRxiv. 2023 Feb 21:2023.02.20.529031. Preprint. [Version 1] doi: 10.1101/2023.02.20.529031 (PMC9980007; doi:10.1101/2023.02.20.529031)

bioRxiv preprint doi: https://doi.org/10.1101/2023.02.20.529031; this version posted February 21, 2023. The copyright holder for this preprint (which was not certified by peer review) is the author/funder, who has granted bioRxiv a license to display the preprint in perpetuity. It is made available under aCC-BY 4.0 International license.

Supplemental Figure Legends

Fig. S1. Competition tests of the T6SS producer strain and the T6SS resistant strain

bioRxiv preprint doi: https://doi.org/10.1101/2023.02.20.529031; this version posted February 21, 2023. The copyright holder for this preprint (which was not certified by peer review) is the author/funder, who has granted bioRxiv a license to display the preprint in perpetuity. It is made available under aCC-BY 4.0 International license.

Strains used for the growth experiments were evaluated for their T6SS-dependent competitive phenotype through (**a**) interspecific co-culture competition against a natural Sensitive strain *(Bacteroides thetaiotaomicron*) (BT) or (**b**) tested for sensitivity to T6SS-dependent intoxication through intraspecific competition against the WT Producer strain. Competitive index values for co-culture competitions used to evaluate T6SS activity in (**a**) reflect *B. fragilis*:*B. thetaiotaomicron* ratios. Competitive index values for co-culture competitions used to evaluate sensitivity to T6SS activity in (**b**) reflect Resistant:Producer or Sensitive:Producer ratios. Mann-Whitney tests used to compare mean competitive index of Producer (WT) vs BT **(a)** or Resistant(Δ*tssC*) vs Producer across conditions (*P<0.05). Results are from four independent biological replicates each with three technical replicates. Data show the mean values ±SD.

Fig. S2. Liquid growth assays involving different mono-, di-, or polysaccharides as carbon sources. The growth of T6SS Producer (WT) and Resistant strains (Δ*tssC*) in defined minimal media supplemented with different carbon sources: D-glucose, D-fructose, D-galactose, D-xylose, sucrose, potato starch, glycogen, and inulin. Mann-Whitney test showed no difference in means between the different strains at each timepoint. For each experiment, the results are from three biological replicates that are the mean of eight technical replicates. Data show the mean ±SD.

Fig. S3. Taxonomic profiling of microbiota from two-strain co-colonization experiments in mice. Taxonomic profiling of the gut microbiota via 16S rRNA amplicon sequencing after 6 months of co-colonization of mice by the Producer strain with a Resistant strain either (**a-b**) lacking *tssC-B-clpV* genes or (**c-d**) a Resistant strain lacking *tssC* only. Taxonomic profiles are shown at the genus level (**a, c**) for taxa within the order Bacteroidales or the order level (**b, d**) for all taxa.

Fig. S4. Quantification of strain relative abundance along the gastrointestinal tract from co-colonization of mice.
The percent of Producer compared to the total of Producer and Resistant strains (Δ*tssBC-clpV*) detected along the GI tract at day 187 after gavage (SI: Small Intestine; CE:Cecum, Col:Colon, Pel:Pellet), calculated by qPCR targeting unique barcodes. One-way ANOVA analysis showed no difference between mean strain abundances across different intestinal sites (P>0.05).

Fig. S5. Co-culture of T6SS producer and T6SS resistant strains in the mouse gut.
The T6SS producer (WT) and T6SS resistant strain (Δ*tssC)* were inoculated in a 1:1 ratio in mice pre-treated to eradicate the endogenous gut microbiota. **(a)** We followed the two strains progression for 180 days (around 6 months) by sampling the mice pellet multiple times. The T6SS producer percentage is shown with a mean of eight. Kruskal-Wallis test showed the difference between sampling at day 1 (one day after gavage) and all subsequent sampling. *P < 0.05 (**b**) T6SS Producer percentage follow-up of each mouse. (**c**) Percentage of gDNA producers detected along the GI tract at day 180 after gavage (SI: Small

bioRxiv preprint doi: https://doi.org/10.1101/2023.02.20.529031; this version posted February 21, 2023. The copyright holder for this preprint (which was not certified by peer review) is the author/funder, who has granted bioRxiv a license to display the preprint in perpetuity. It is made available under aCC-BY 4.0 International license.

Intestine, CE:Cecum, Col:Colon, Pel:Pellet). Kruskal-Wallis showed the absence of difference between the different localization (P>0.05).

Fig. S6. Producer strains do not lose T6SS activity following extended co-colonization of mice. WT clones were isolated from the last fecal pellets of the two six months experiment (Fig.2 and Fig.S5). Screening for the absence of Hcp secretion was performed but none of the clones showed a loss of secretion. (**a**) Three clones were randomly chosen, and detection of secreted Hcp is shown by an ELISA using Hcp-specific antisera. Mann-Whitney test was used to compare parental WT strain to all other strains (*P<0.05). Results are from five biological replicates. (**b**) T6SS activity was further tested for the same isolates used in (**a**) via two-strain co-culture growth assays using *B. thetaiotaomicron* (BT) as a susceptible target strain. Mann-Whitney test compare WT vs BT against all the other conditions (*P<0.05). Data points represent the mean of three technical replicates for each of four biological replicates ±SD.

Fig. S7. Quantification of strain relative abundance along the gastrointestinal tract from three-strain co-colonization of mice.
The T6SS Producer (WT), Resistant strain (Δ*tssBC-clpV)*, and Sensitive strains (Δ*E-I*Δ*tssBC-clpV*) were inoculated in a 1:1:1 ratio in mice pre-treated to eradicate the endogenous gut microbiota. After 88 days, the contents of the small intestine (SI), cecum (CE), colon (Col), were collected upon sacrifice. The mean percent relative abundance of Producer (**a**), Resistant (**b**), and Sensitive (**c**) strain by quantification of barcodes via qPCR is shown for each mouse individually over time. D'Agnostino & Pearson tests were used to determine normal distribution of data and one-way ANOVA (for Resistant strain) and Kruskal-Wallis (for Producer and Sensitive strains) tests indicated that no significant differences between strain composition across intestinal cites.

Supplementary Table 1
Classification of adult gut metagenomes by *B. fragilis* GA3 T6SS status and taxonomic relative abundances for species identified to be associated with GA3 via PhILR.
Supplementary Table 2
Reference genomes of *B. fragilis* annotated by GA3 status.
Supplementary Table 3
Genomic differences identified in *B. fragilis* Producers evolved in mice co-colonized with Resistant strains.
Supplementary Table 4
Strains, plasmids, and primers used in this study.

bioRxiv preprint doi: https://doi.org/10.1101/2023.02.20.529031; this version posted February 21, 2023. The copyright holder for this preprint (which was not certified by peer review) is the author/funder, who has granted bioRxiv a license to display the preprint in perpetuity. It is made available under aCC-BY 4.0 International license.

*

Fig. S1

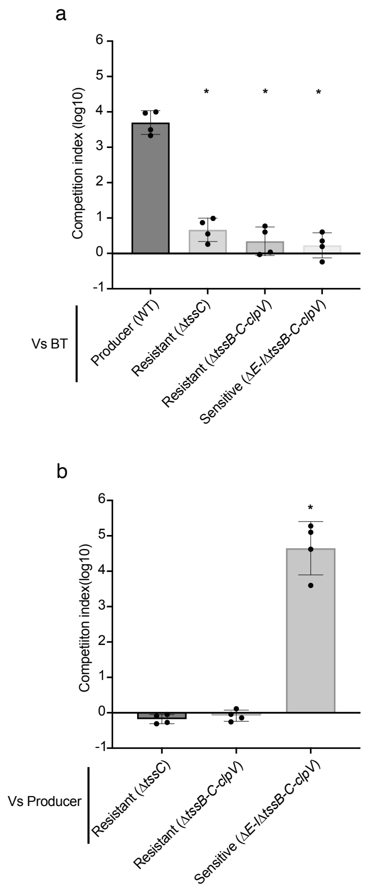

bioRxiv preprint doi: https://doi.org/10.1101/2023.02.20.529031; this version posted February 21, 2023. The copyright holder for this preprint (which was not certified by peer review) is the author/funder, who has granted bioRxiv a license to display the preprint in perpetuity. It is made available under aCC-BY 4.0 International license.

Fig. S2

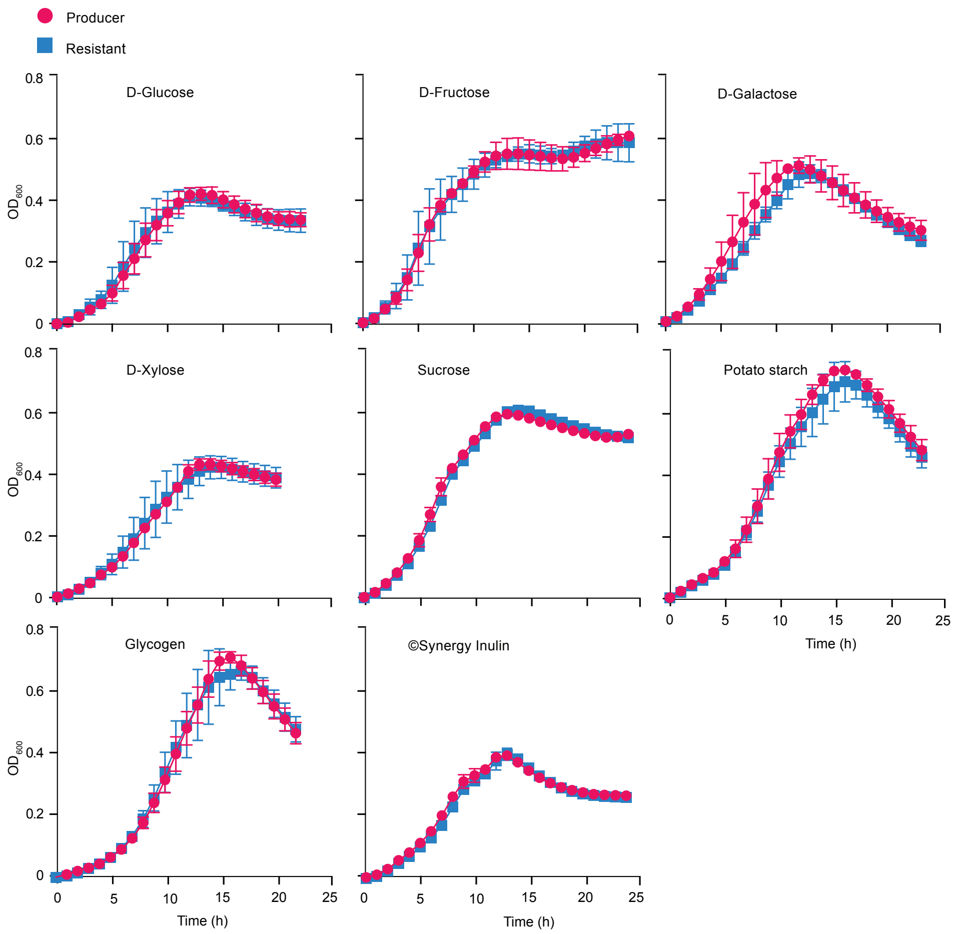

bioRxiv preprint doi: https://doi.org/10.1101/2023.02.20.529031; this version posted February 21, 2023. The copyright holder for this preprint (which was not certified by peer review) is the author/funder, who has granted bioRxiv a license to display the preprint in perpetuity. It is made available under aCC-BY 4.0 International license.

Fig. S3

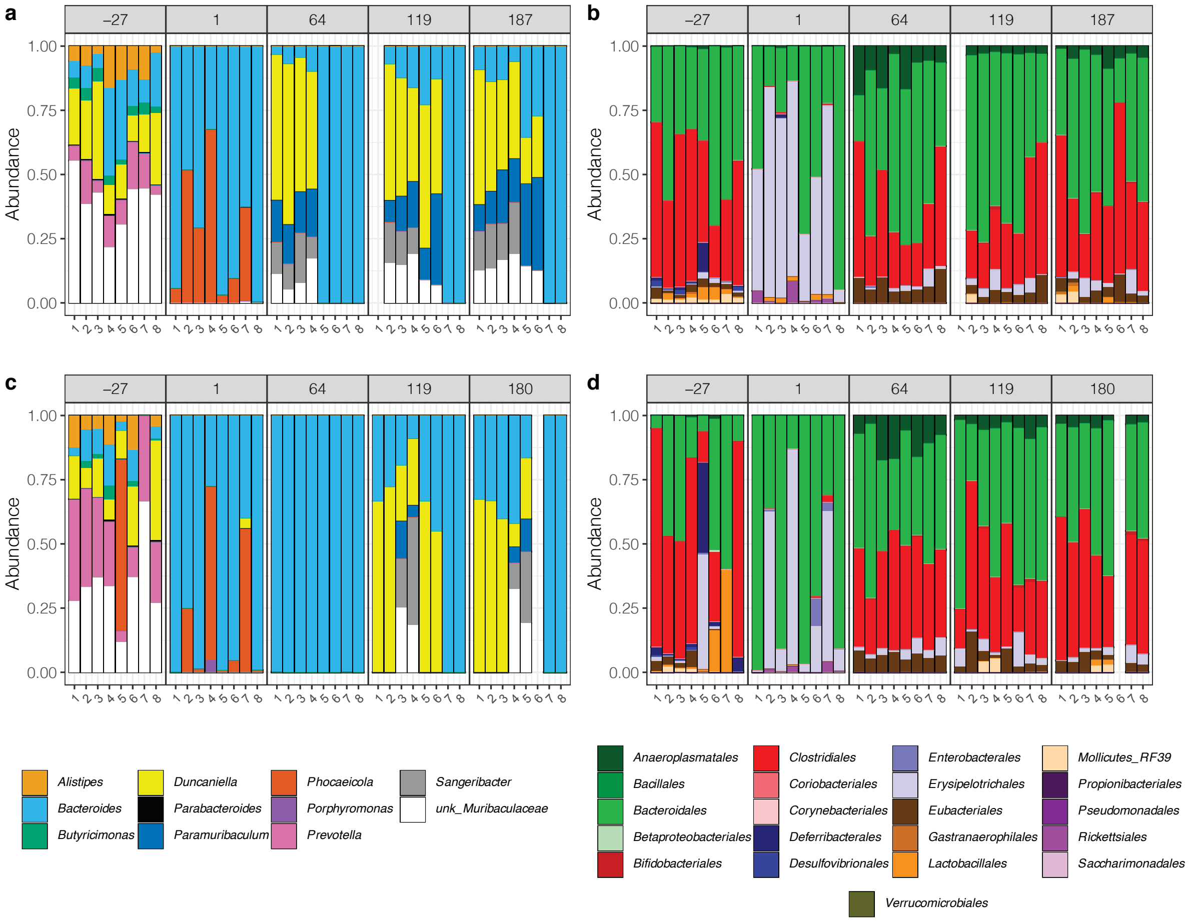

bioRxiv preprint doi: https://doi.org/10.1101/2023.02.20.529031; this version posted February 21, 2023. The copyright holder for this preprint (which was not certified by peer review) is the author/funder, who has granted bioRxiv a license to display the preprint in perpetuity. It is made available under aCC-BY 4.0 International license.

Fig. S4

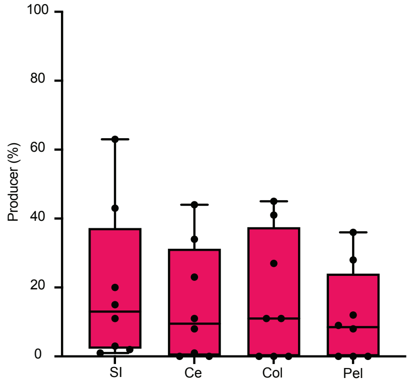

bioRxiv preprint doi: https://doi.org/10.1101/2023.02.20.529031; this version posted February 21, 2023. The copyright holder for this preprint (which was not certified by peer review) is the author/funder, who has granted bioRxiv a license to display the preprint in perpetuity. It is made available under aCC-BY 4.0 International license.

Fig. S5

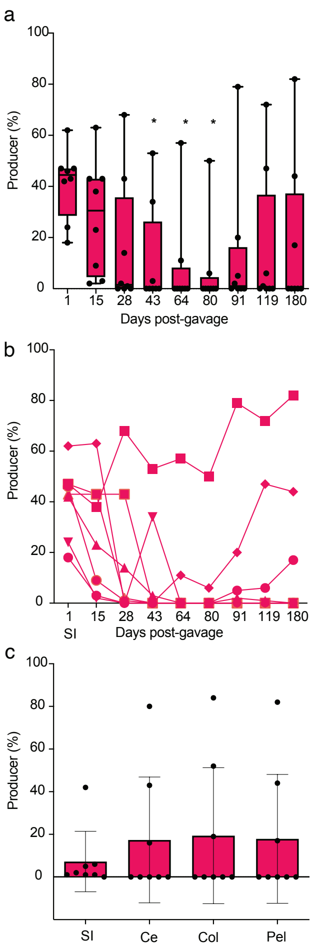

bioRxiv preprint doi: https://doi.org/10.1101/2023.02.20.529031; this version posted February 21, 2023. The copyright holder for this preprint (which was not certified by peer review) is the author/funder, who has granted bioRxiv a license to display the preprint in perpetuity. It is made available under aCC-BY 4.0 International license.

Fig. S6

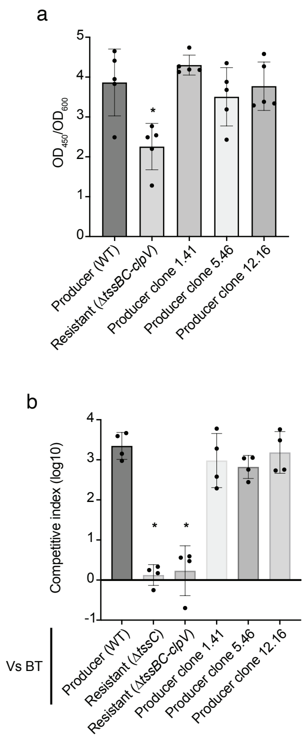

bioRxiv preprint doi: https://doi.org/10.1101/2023.02.20.529031; this version posted February 21, 2023. The copyright holder for this preprint (which was not certified by peer review) is the author/funder, who has granted bioRxiv a license to display the preprint in perpetuity. It is made available under aCC-BY 4.0 International license.

Fig. S7

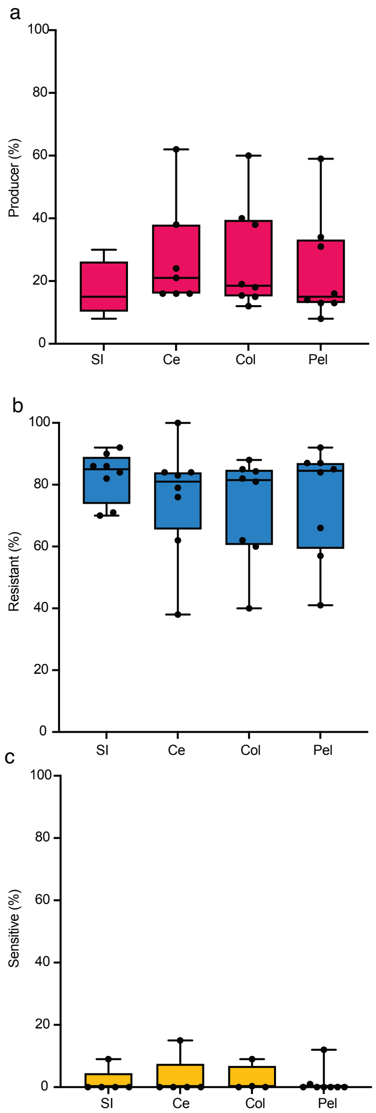

Supplement: Supplement 2 [file NIHPP2023.02.20.529031v1-supplement-2.pdf]
